# Editorial for the Special Issue “Cardiometabolic Health in Relation to Diet and Physical Activity: Experimental and Clinical Evidence”

**DOI:** 10.3390/nu15132903

**Published:** 2023-06-27

**Authors:** Abeer M. Mahmoud

**Affiliations:** 1 Division of Endocrinology, Diabetes, and Metabolism, Department of Medicine, College of Medicine, The University of Illinois at Chicago, Chicago, IL 60612, USA; amahmo4@uic.edu; 2 Department of Kinesiology and Nutrition, College of Applied Health Sciences, The University of Illinois at Chicago, Chicago, IL 60612, USA

This Special Issue seeks to compile a centered, influential, and well-referenced volume on the impact of diet and physical activity on various cardiometabolic risk factors. It is anticipated that the global prevalence of cardiometabolic illness, now a major health and economic burden, will continue to climb. There is mounting evidence that excessive caloric intake, poor food choices, and inactivity all contribute to the development of cardiometabolic risk factors such as altered glucose metabolism, insulin resistance, dyslipidemia, and hypertension [1]. This Special Issue aims to present robust data from recent experimental and observational research to establish the link between lifestyle variables such as diet and physical activity and cardiometabolic risk. This issue also includes studies that analyze the related molecular and subcellular pathways that mediate the link between these lifestyle factors and cardiometabolic risk.

Public health initiatives that promote healthy lifestyles, such as eating well and regularly exercising, are warranted in light of the persistently high prevalence of cardiometabolic risk factors. This issue features cutting-edge scientific assessments of food and lifestyle impacts on cardiometabolic health from eminent experts such as (1) the role of a healthy lifestyle in reducing stroke recurrence, (2) cultural preferences for healthy living and activities, (3) the impact of engaging in risky health behaviors in inducing cardiometabolic risk in Latinos, (4) the efficacy of web-based interventions in promoting a healthy lifestyle, (5) oral health as a predictive factor of eating preferences and hypertension in elderly, (6) the effectiveness of a combined citrulline and antioxidant supplements in improving endothelial function in postmenopausal women, (7) the acute effect of fiber-rich supplements in improving postprandial glucose and insulin levels, (8) fecal microbiota capsules in inducing bile acid metabolism and metabolic function, (9) the role of salt sensitivity genetic traits in high-fat diet-induced hypertension, and (10) the effectiveness of fasting interventions in preventing metabolic diseases.

Stroke continues to be the leading cause of disability and death worldwide, placing a significant strain on healthcare systems across the globe. Hypertension and oxidative stress play an essential role in the pathophysiology of stroke. In a cross-sectional study, Pham et al. [2]. investigated the moderating influence of antioxidative lifestyle factors in the link between hypertension and stroke recurrence. The authors of this study determined the ratio between the prooxidant capacity derived from smoking, drinking, being overweight, and obesity and the antioxidant capacity presented by a healthy diet that includes fruit and vegetable consumption and physical exercise in 951 stroke patients. In hypertensive individuals, a higher antioxidant/prooxidant ratio was associated with a decreased risk of stroke recurrence (odds ratio = 0.83; *p* = 0.022). This study supports the growing evidence that 75% of stroke cases can be prevented by adopting a healthy lifestyle [3,4].

It is important to note that some ethnic groups are more susceptible to cardiometabolic diseases than others. For instance, Latinxs are disproportionately affected by cardiometabolic conditions. Therefore, it is imperative to focus on the culture-related behaviors and modifiable risk factors that place this population at high risk for cardiovascular and metabolic diseases. In a unique study by Sanchez-Johnsen et al. [5], the cultural preferences of the Latin population in Chicago were tested regarding healthy lifestyle factors such as diet and physical activity. Interviews with 203 Puerto Ricans and Mexicans demonstrated that the majority (60–80%) of the Latin population preferred interventions that are related to their culture, either the language used to communicate with them, exercise programs that include Latin or salsa movements or salsa music, dichos (Latino sayings), or cuentos (folktales or stories). The results suggest that therapies targeting one’s way of life and perception of one’s body image may reduce the risk of cardiometabolic illnesses.

Dr. Sanchez-Johnsen’s research team also examined the role of engaging in multiple risky health behaviors in inducing cardiometabolic risk in Latinos. The team collected data on three health behaviors: (1) nutrition pattern, including vegetable and fruit intake; (2) physical activity; and (3) smoking. The study included 104 Puerto Rican and 99 Mexican men who reported health risk behaviors, 5% reported none, 30% reported one, 47% reported two, and 18% reported all three. These results emphasize the need to develop lifestyle interventions for Latinos that address various health risk behaviors associated with cardiometabolic diseases [6].

One of the lessons that were learned during the COVID-19 era is that remote interventions are needed that can access diverse populations, surmount limitations and barriers, and provide efficient approaches for promoting healthy behaviors. In their “Living Better” initiative, Mzquiz-Barberá et al. [7] implemented a self-administered web-based intervention to enhance lifestyle and assess the impact of this enhancement on long-term health benefits in obese patients with hypertension. The initiative also looked at the results of repeating the intervention during the COVID-19 epidemic (reintervention). All advantages gained in response to the “Living Better” program, such as reduced systolic and diastolic blood pressure, decreased body weight and body mass index, and improved dietary and physical activity behavior, were maintained throughout the duration of the study (3 years). These parameters also saw significant improvement in response to repeating this intervention during the COVID-19 pandemic.

In addition to nutrition and physical activity, oral health’s contribution to overall health should not be underestimated. Oral health and tooth integrity significantly impact mastication ability, food selection, nutrition status, and general health, particularly in older adults. Marito et al. [8] examined the dietary preferences and patterns of 894 adults over 65 to comprehend the link between oral condition and hypertension in this geriatric population. Specifically, they sought to determine how these dietary preferences and patterns may explain the association between oral health and hypertension. Dietary intake was assessed using food frequency questionnaires, and oral health was evaluated by determining the number of teeth, the force of teeth closure, posterior occlusal support, mastication efficiency, oral hydration, and oral bacterial level. In addition to body mass index and age, inadequate posterior occlusion positively predicted hypertension (odds ratio = 1.72). The latter was also associated with a lack of fruit and vegetable consumption, indicating that oral health condition significantly impacts dietary preferences and, consequently, malnutrition-related illnesses such as hypertension.

Increasing research suggests that using dietary supplements may improve cardiometabolic health. Arginine was identified as one of the nutritional components that enhances vascular function; nevertheless, its oral administration is ineffective due to its degradation via arginase. Citrulline, unlike arginine, is not degraded by arginase and was reported to enhance plasma arginine and arterial elasticity and reduce resting blood pressure in middle-aged and elderly individuals [9]. Therefore, Figueroa et al. [10] tested the effect of four weeks of oral citrulline with and without the antioxidant glutathione on the vascular function of postmenopausal women, a population susceptible to endothelial dysfunction associated with deficiency of arginine due to oxidative stress. While citrulline administration increased arginine levels, only the combination of citrulline with the antioxidant glutathione improved vasoreactivity of the brachial artery and attenuated the vasoconstriction in response to sympathoexcitation via the cold press test.

Incorporating more dietary fiber into one’s diet has been shown to improve health and lower the likelihood of developing certain metabolic illnesses. Hammad Ullah et al. [11] measured the effect of a single meal rich in fibers on postprandial glucose and insulin levels. In this study, 40 normoglycemic participants with mild insulin resistance were randomized into two groups that received brewer’s spent grain (BSG) extract-based food supplement or placebo with the provided breakfast meal. The BSG food supplement was rich in β-glucan, arabinoxylans, resistant starch, and other soluble fibers. After 120 min after the meal, the postprandial blood glucose and insulin values in the treatment group were considerably lower than the corresponding values in the placebo group. This study demonstrated the acute effect of a fiber-rich supplement in restoring postprandial blood glucose and insulin levels to those at baseline in individuals with modest insulin resistance. Further research is required to confirm that this acute effect is translated into long-term benefits, which will result in guidelines for supplementing the diets of individuals with mild insulin resistance with fiber.

Fecal microbiota is considered a novel direction in the field of supplementation, particularly concerning their effects on endogenous critical pathways such as bile acid metabolism. Bile acids are a family of bioactive metabolites whose function is mediated via bile acid receptors to promote metabolic health. The research conducted in the laboratory of Dr. Cummings suggests a therapeutic function for fecal microbiota transplantation in metabolic diseases by enhancing bile acid metabolism. In a double-blind, randomized study, obese patients treated with fecal microbiota capsules derived from a lean, healthy volunteer demonstrated improved gut bacterial bile acid metabolism and metabolic parameters. These findings were supplemented with analyses of stool specimens from respective patients to examine the microbial candidates that contribute the most to the metabolic improvement and the enhancement of gut bacterial bile acid metabolism. Several candidate bacteria that may contribute to the metabolic benefits of fecal microbiota transplantation and intestinal bacterial bile acid metabolism were identified in this research and stand as a valuable source for future research by the same group and others [12].

A relatively new area of study, nutrigenomics, focuses on the reciprocal relationship between what we eat and how our DNA interprets that food. In this special issue, original work by Lee et al. [13] showed that the same genetic traits that determine salt sensitivity may also influence the susceptibility to high-fat diet-induced hypertension. In this 12-week study, Dahl salt-resistant and salt-sensitive rats were administered either a high-fat diet (60% fat) or a chow diet (8% fat). Hypertension was observed in the salt-sensitive rodents but not the salt-resistant rats, even though both groups gained equal amounts of weight. In this research, genes implicated in adipogenesis, tissue remodeling, inflammation, and vascular function were analyzed in response to a high-fat diet in salt-sensitive and salt-resistant mouse models.

Finally, the current issue includes a comprehensive review article by Vrdoljak et al. [14] which summarizes the preclinical and clinical evidence regarding the impact of various forms of fasting on metabolic syndrome. Intermittent fasting and time-restricted feeding are two fasting interventions discussed in this article. This article discusses the inconsistent efficacy of these interventions in inducing tangible cardiometabolic benefits in humans relative to animal studies and the molecular and subcellular events that may have mediated the observed changes in the trials.

In conclusion, research that is now being conducted into the function of diet, exercise, and other lifestyle variables is continually attempting to shed light on the importance of these factors in sustaining cardiometabolic health. In addition, ongoing research is aiming to determine the molecular pathways that mediate the connection between one’s lifestyle and current health state. In spite of this, there is a growing need for more clinical, translational, and mechanistic research to address outstanding issues about the discrepancy in results seen across various populations and between human and animal studies.
